# Stargardt macular dystrophy and therapeutic approaches

**DOI:** 10.1136/bjo-2022-323071

**Published:** 2023-11-08

**Authors:** Kaoru Fujinami, Nadia Waheed, Yannik Laich, Paul Yang, Yu Fujinami-Yokokawa, Joseph J Higgins, Jonathan T Lu, Darin Curtiss, Cathryn Clary, Michel Michaelides

**Affiliations:** 1 Laboratory of Visual Physiology, Division of Vision Research, National Institute of Sensory Organs, NHO Tokyo Medical Center, Meguro-ku, Tokyo, Japan; 2 Institute of Ophthalmology, University College London, London, UK; 3 Moorfields Eye Hospital NHS Foundation Trust, London, UK; 4 Department of Ophthalmology, Tufts University School of Medicine, Boston, Massachusetts, USA; 5 Eye Center, Medical Center, University of Freiburg Faculty of Medicine, Freiburg, Germany; 6 Oregon Health and Science University Casey Eye Institute, Portland, Oregon, USA; 7 Department of Health Policy and Management, Keio University School of Medicine Graduate School of Medicine, Shinjuku-ku, Tokyo, Japan; 8 SalioGen Therapeutics Inc, Lexington, Massachusetts, USA; 9 Applied Genetic Technologies Corporation, Alachua, Florida, USA

**Keywords:** Genetics, Retina, Imaging, Treatment other, Electrophysiology

## Abstract

Stargardt macular dystrophy (Stargardt disease; STGD1; OMIM 248200) is the most prevalent inherited macular dystrophy. STGD1 is an autosomal recessive disorder caused by multiple pathogenic sequence variants in the large *ABCA4* gene (OMIM 601691). Major advances in understanding both the clinical and molecular features, as well as the underlying pathophysiology, have culminated in many completed, ongoing and planned human clinical trials of novel therapies.

The aims of this concise review are to describe (1) the detailed phenotypic and genotypic characteristics of the disease, multimodal imaging findings, natural history of the disease, and pathogenesis, (2) the multiple avenues of research and therapeutic intervention, including pharmacological, cellular therapies and diverse types of genetic therapies that have either been investigated or are under investigation and (3) the exciting novel therapeutic approaches on the translational horizon that aim to treat STGD1 by replacing the entire 6.8 kb *ABCA4* open reading frame.

## Introduction

Stargardt macular dystrophy or Stargardt disease (STGD1; OMIM: 248200) is one of the most common macular dystrophies.^1–8^ STGD1 was first described by Karl Stargardt in 1909 and is characterised by bilateral progressive loss of visual acuity (VA) and central vision.^9^ There are three presentations of STGD1, childhood onset, adulthood onset and late onset, with earlier presentation being associated with a worse prognosis.^3 7 10–20^

**Figure 1:**
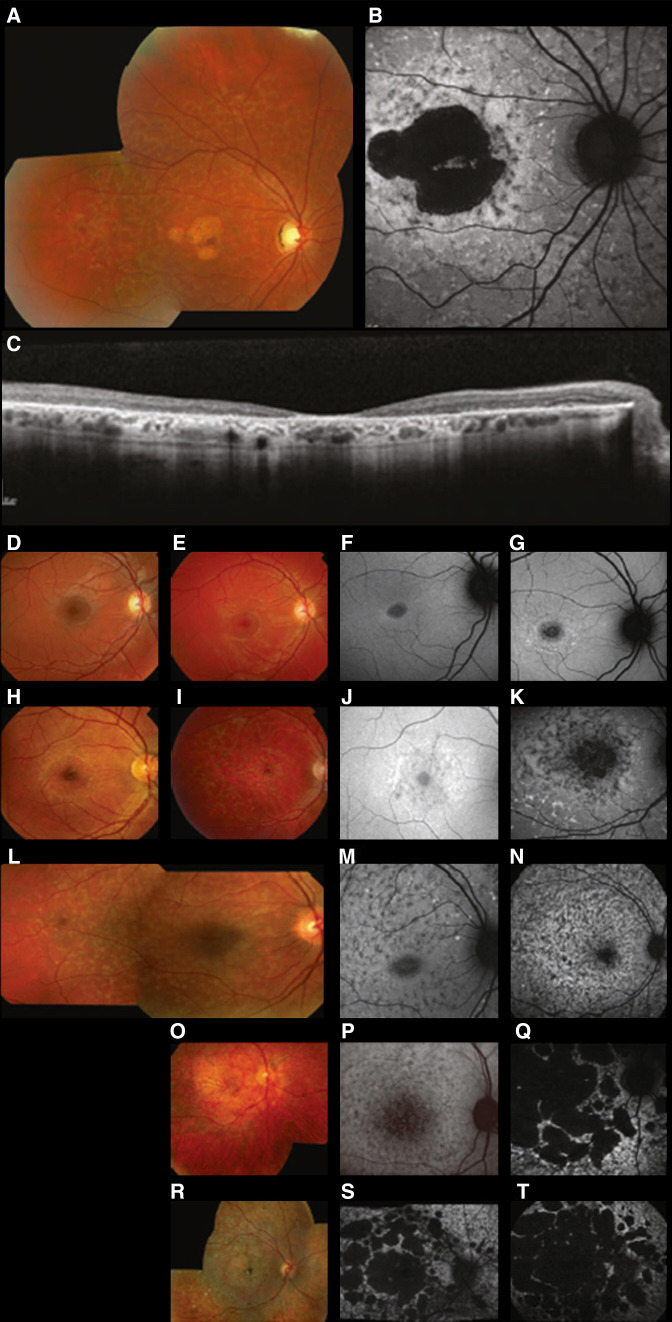
Representative cases of Stargardt disease (STGD1). Typical findings of Stargardt disease (STGD1; A–C). Fundus photograph of the right eye showing macular atrophy with yellow-white flecks at the level of the retinal pigment epithelium (RPE; A). Fundus autofluorescence (FAF) imaging identified an area of decreased autofluorescence (DAF) at the macula and multiple surrounding foci of abnormal AF (B). Spectral-domain optical coherence tomography (SD-OCT) demonstrated marked loss of outer retinal layers and RPE at the macula, with multiple hyper-reflective foci corresponding to flecks (C). A broad range of FAF patterns and progression over time in STGD1 are presented, with corresponding fundus photographs (D–T). FAF pattern can be classified into three types based on the area(s) of DAF and the background features (heterogeneous or homogeneous): type 1 (Baseline; F) to type 2 (follow-up; G), type 1 (baseline; J) to type 2 (follow-up; K), type 2 (baseline; M) to type 2 (follow-up; N), type 2 (baseline; P) to type 3 (follow-up; Q), type 3 (baseline; S) to type 3 (follow-up; T). Genetic information (*ABCA4*, Transcript ID: NM_000350.3; ENST00000370225.4): Case 1 (top row; : c.4139C>T, p.Pro1380Leu. Case 2 (second row; D–G): c.3322C>T, p.Arg1108Cys; c.6079C>T, p.Leu2027Phe. Case 3 (third row; H–K): c.768G>T; c.2588 G>C, p.Gly863Ala. Case 4 (fourth row;I–N): Unavailable. Case 5 (fifth row; O–Q): c.1622T>C, p.Leu541Pro; c.3113C>T, p.Ala1038Val; c.617_618delCG, p.Ser206ArgfsTer320. Case 6 (bottom row; R–T): c.5461–10T>C. *Permission to reuse the figure for publication in the journal has been obtained by the licensed content publisher, Springer Nature (Number: 5415100406042; License date: 23 October 2022).

STGD1 typically presents with a variable degree of macular atrophy and yellow-white flecks at the level of the retinal pigment epithelium (RPE) (figure 1).^8 10 11 14 21^ However, there are a broad range of manifestations resulting in a large spectrum of clinical presentations, onset, progression, psychophysical and electrophysiological findings, as well as variable prognosis.^6 10–13 15–20 22–32^

The global prevalence of STGD1 has been estimated at 1 per 6578.^33^ Due to the progressive nature and often early onset of STGD1, patients typically face long-term health-related financial, emotional and psychological implications. Although information on the economic burden of STGD1 alone is not available, these impacts have been studied in a broad range of inherited retinal diseases (IRDs).^34^ Some studies estimate the total cost is over US$27.5 billion per year among people aged 40 years and younger with eye disorders.^35 36^

In 1997, disease-causing sequence variants in the *ABCA4* (ATP binding cassette subfamily A member 4; OMIM: 601691) gene were identified as the cause of STGD1^37^; with more than 2000 variants found to date. The carrier frequency for a disease-causing variant in *ABCA4* may be as high as 1:20; although the true prevalence of retinopathy attributed to *ABCA4* variants is likely much higher than that of STGD1, given it can also cause other phenotypes including cone dystrophy, cone-rod dystrophy and rod-cone dystrophy.^1 3 6 7 22 38^ Moreover, the bone spicule pigmentation of *ABCA4*-associated retinopathy attributed to severe cone-rod dystrophy (formerly retinitis pigmentosa inversa) is now widely accepted as a sign of late-stage cone-rod dystrophy and peripheral degeneration, rather than rod-cone dystrophy.^39^

An increasing amount of research on the clinical and molecular genetics of STGD1/*ABCA4* has been performed over the past 15 years. This has facilitated a growing understanding of the underlying pathophysiology, which has resulted in both completed and ongoing trials, as well as a broad range of planned clinical trials.^6 7 40–42^ Many types of interventions have been explored to treat STGD1, including pharmacological treatments, regenerative cell therapies^43 44^ and gene replacement/supplementation therapy.^45 46^ Increasingly, precision medicine focusing on particular variants and mechanisms has been gaining attention (including gene editing).^7 42 47^

The aim of this review is to describe the phenotypic and genotypic characteristics, imaging findings, natural history and pathogenesis of the disease. Additionally, the characteristics of particular *ABCA4* variants, a pathogenicity assessment and a concise overview of the therapeutic landscape—past, present and future—will be presented.

## Disease overview

### Gene family/gene function

**Figure 2:**
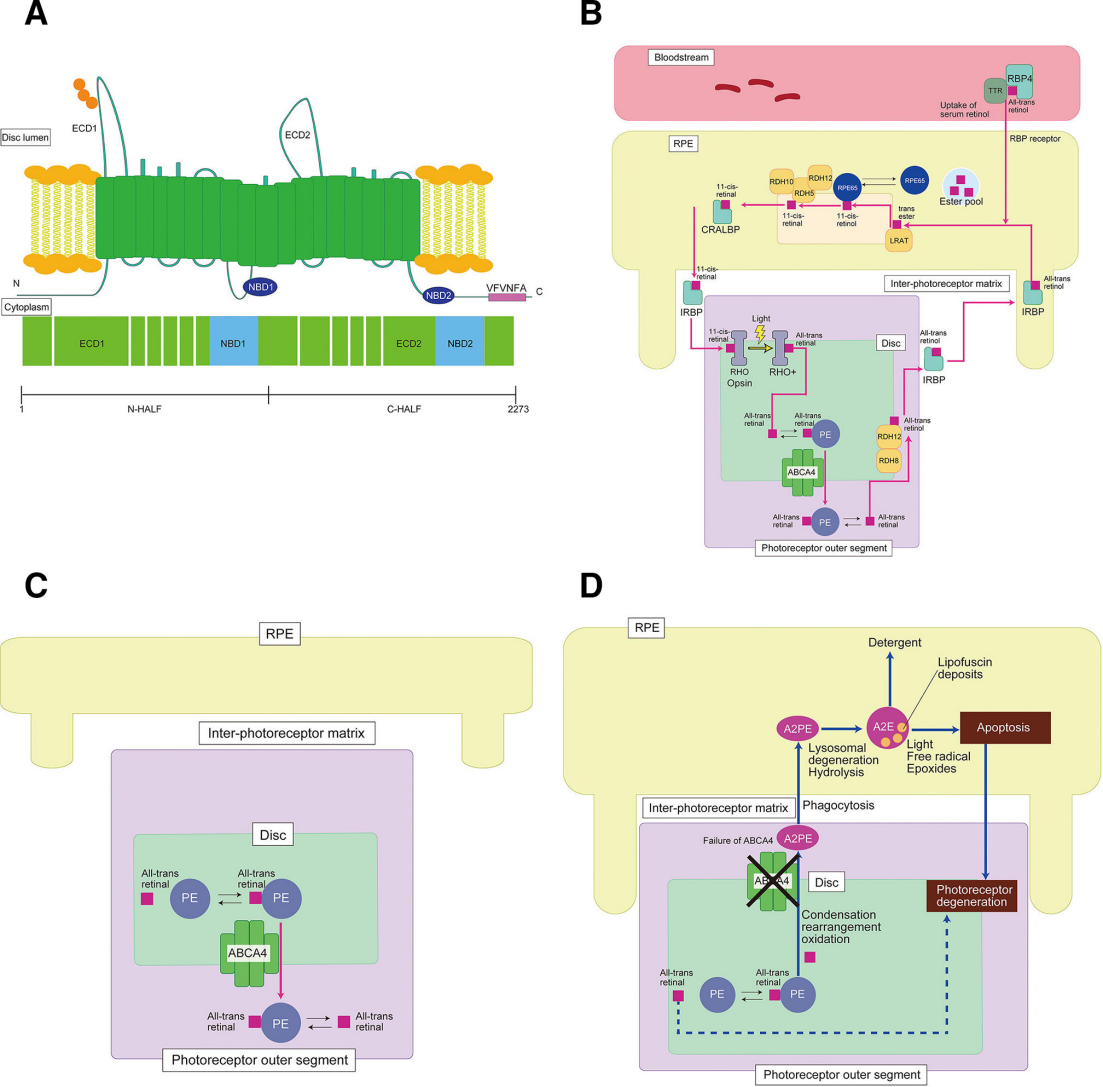
Molecular mechanisms of STGD1 (*ABCA4*-retinopathy) a schematic of ABCA4 protein structure (A), the visual cycle (B), transport (C) and failure of transport leading to retinal degeneration (D). The *ABCA4* gene transcribes a large retina-specific ABCA4 protein with two transmembrane domains (TMD), two glycosylated extracellular domains (ECD) and two nucleotide-binding domains (NBD) (A). All-trans retinal is released from the light-activated rhodopsin/cone opsin into the rod/cone outer segments (B) to form a complex with phosphatidylethanolamine (PE), resulting in N-ret-PE, then this complex is actively transported to the disc surface by ABCA4 (C). Failure of this transport results in accelerated deposition of a major lipofuscin fluorophore (A2E) in the RPE, which causes RPE dysfunction and cell death, with subsequent photoreceptor cell loss over time (D).

The *ABCA4* gene is a large, highly polymorphic gene with an estimated size of 6819 bp encoding a 2,273-amino acid protein, including 50 exons.^37^ *ABCA4*, formerly described as *ABCR*, is a member of the ABC transporter gene superfamily, encoding the retinal-specific transmembrane protein, a member of the ATP-binding cassette transporter superfamily.^3 6 48^ ABCA4 contains two transmembrane domains, two glycosylated extracellular domains and two nucleotide-binding domains (figure 2A).^3 6 48^

ABCA4 is localised along the rim of the rod/cone outer segment discs and is involved in the active transport of retinoids from photoreceptor to RPE in the retinoid cycle.^48–51^ The visual/retinoid cycle involves a series of enzyme-catalysed reactions that convert all-trans retinal, generated with photobleaching of rhodopsin/cone opsin, back to 11-*cis* retinal.^48 50–53^ All-trans retinal is released from the light-activated rhodopsin/cone opsin into the rod/cone outer segments to form a complex with phosphatidylethanolamine (PE), resulting in N-retinylidene-PE (N-ret-PE).^6 48 54^ This complex is then actively transported to the disc surface by ABCA4 (figure 2B,C). ABCA4 has also been shown to be expressed at lower levels in the RPE, where it may serve a similar function for the recycling of retinaldehydes.^54^

### Molecular genetics

The vast allelic heterogeneity of *ABCA4* is clearly demonstrated by the number of reported sequence variations (>2000) to date, resulting in macular dystrophy, cone dystrophy, cone–rod and rod–cone dystrophy.^3 6–8^ Due to this heterogeneity, establishing genotype–phenotype correlations is highly challenging. Likewise, the identification of *ABCA4* genetic characteristics related to intronic variants remains largely elusive, despite genetic sequencing advances. Deep intronic variants have been shown to significantly account for the missing heritability in STGD1 and have been associated with late-onset disease and mild phenotype.^55–57^ However, due to the highly polymorphic nature of the *ABCA4* gene, the genetic and pathogenic features of deep intronic variants remain difficult to characterise.

Null variants or variants predicted to be more deleterious are generally associated with earlier onset disease and characterised by a more severe, rapidly progressive phenotype, often with more generalised retinal involvement.^6 8 11–13 16 25 26 32^ Milder variants, such as missense variants, are often associated with later onset disease, typically milder, more slowly progressive and more likely isolated to the macula.^58^ Although certain missense variants can produce severe functional effects similar to nulls (eg, p.Leu541Pro/p.Ala1038Val (complex), p.Glu1022Lys, p.Cys1490Tyr, p.Glu1087Lys, p.Thr1526Met, p.Arg1640Trp and p.Cys2150Tye p.Cys2150Tyr).^13 16 25 26 58^ The interaction between the variants (including disease-causing and benign variants) may also affect function.^59^ Nevertheless, certain missense variants, including p.Arg2030Gln, are commonly observed in the mildest *ABCA4*-associated phenotype, late-onset/foveal sparing STGD1 (FS-STGD1).^14 26^

While *ABCA4* allelic heterogeneity is high, there are founder variants associated with STGD1 in various racial and ethnic groups as well as differences in clinical features related to *ABCA4*-retinopathy.^6^ There have been larger cohort STGD1 studies featuring the genotypic profile and phenotypic correlations for the White populations in European/North American, although there are a limited number of studies for the Latin, Asian, African and other populations.^5 8 32 60 61^ However, further studies are required to better understand the clinical difference and effects across different ethnic and racial groups.

A category of rare hypomorphic alleles has also been characterised, which are typically observed in milder phenotypes with better prognosis.^62^ Lee *et al* showed that these hypomorphic variants can modulate the severity of the phenotype irrespective of the severity of the allele in trans.^62^ Notably, the mechanism of hypomorphic alleles or milder variants has been attributed to either reduced function of the ABCA4 protein produced in normal amounts (ie, missense variants) or reduced production of a normal functioning protein (ie, splice variants). The aberrant splicing in the *ABCA4* gene and resulting variants, whose pathogenicity was previously unknown, has more recently been reclassified as pathogenic based on midi-gene and fibroblast assays.^63 64^

### Molecular mechanisms

Failure of transport due to ABCA4 dysfunction or mislocalisation leads to the inefficient removal of N-ret-PE from photoreceptor outer segments, resulting in an accumulation of bisretinoid compounds in the outer segment discs and ultimately in toxic levels of bisretinoid A2PE in photoreceptor membranes.^48 49^ A2PE is hydrolysed to form the highly toxic metabolite N-retinylidene-N-retinyl-ethanolamine (A2E), which accumulates as a major component of lipofuscin in RPE cells, and ultimately causes RPE dysfunction and death, with subsequent photoreceptor dysfunction/loss (figure 2D).^53^

Previous studies of STGD1 mouse models (ie, *ABCA4* knockout) support the aforementioned pathogenesis; however, there are limitations such as lack of a macula in mice and the mild phenotype in mouse models showing a later-onset disease with slower degeneration than that of typical patients with STGD1.^51 65^ Moreover, there is data from multimodal high-resolution imaging studies in humans that in some cases photoreceptor cell loss may precede RPE cell dysfunction/loss.^17 19 20 66 67^

### Clinical aspects

Patients with STGD1 commonly present with progressive bilateral central vision loss. The onset is often in the first or second decades of life.^11 12 24^ The onset relates to the disease severity; an earlier onset disease is associated with more deleterious variants compared with adult-onset disease, which is more frequently due to missense variants.^11–14 16 25–27^

Comprehensive investigations are crucial for clinical diagnosis and monitoring, including fundus photography, fundus autofluorescence (FAF) imaging, spectral-domain optical coherence tomography (SD-OCT) and electrophysiological assessment.^1 3 6 7^ Likewise, clinical classifications are useful to assess the disease severity associated with a particular genotype group.^11 12 19 20 23 25 32^

At an early stage, ophthalmoscopy can reveal a normal retina or minimal retinal abnormalities, including foveal reflex abnormality, white macular dots and RPE disturbance, with or without vision loss.^15–17^ Retinal imaging with FAF, SD-OCT and electrophysiological assessment (including pattern, full-field and multifocal electroretinograms; PERG, FFERG, mfERG) are useful for diagnosis.^42 68–70^ Notably, children with STGD1 may not have retinal flecks on funduscopy or FAF at the early stage, but over time may develop these flecks associated with increasing macular atrophy.^15 16^ In very early childhood-onset disease with relatively preserved vision, macular atrophy involves the parafovea and spares the foveola, and these changes are preceded by fine, symmetrical, yellowish-white dots at the central macula in some cases and/or characteristic loss of outer nuclear layer transparency on SD-OCT.^15–17^

Electrophysiological assessment is particularly helpful in providing better-informed advice on prognosis.^11^ A classification of three functional phenotypes based on electrophysiological findings is well-established: group 1—severe PERG abnormality (macular dysfunction) with normal FFERGs; group 2—severe PERG abnormality with additional generalised cone dysfunction on FFERGs and group 3—severe PERG abnormality with additional generalised cone and rod dysfunction on FFERGs.^11 22^ A longitudinal ERG study has confirmed the prognostic implications of the aforementioned ERG groups, with group 1 having the best prognosis; group 2 having an intermediate or variable prognosis; and group 3 having the worst prognosis.^11^ All patients with initial rod ERG involvement demonstrated clinically significant electrophysiological deterioration; whereas, only 20% of patients with normal FFERGs at baseline showed clinically significant progression over time.^11^ These findings are supported by the association with genotype grouping (eg, group 3 is associated with a greater prevalence of null variants), and are also relevant in the design, patient selection and monitoring of potential therapeutic interventions.^11 16 20^

STGD1 with a later age of onset has been increasingly recognised. Patients with late-onset STGD1 often develop the FS phenotype (FS-STGD1).^10 14 71^ FS-STGD1 thereby is characterised by relatively preserved VA and foveal structure and function in the early and intermediate stages of the disease.^14^ SD-OCT often exhibits outer retinal tubulation at the edge of atrophy, suggesting that the primary site of degeneration of this phenotype is the RPE and choroid.^14^ On the other hand, patients with foveal atrophy can manifest photoreceptor cell loss at the fovea at the early stage. Therefore, the presence of two distinct phenotypes—non-FS-STGD1, which is primarily childhood-onset and adulthood-onset STGD1, and FS-STGD1—suggests more than one disease mechanism in *ABCA4*-associated retinopathy.^14^ The fact that a different distribution of disease-causing variants exists between these two phenotypes appears to support this hypothesis.^14 26^

### Natural history

Natural history studies play a key role in advancing understanding of disease progression.^20 24^ Over the past 8 years, multicentre, international, large-cohort studies (>250 subjects) have been conducted: the retrospective and prospective Natural History of the Progression of Atrophy Secondary to Stargardt Disease (ProgStar) studies.^24^ The aims were to characterise the natural history and identify sensitive, reliable and clinically relevant outcome measures, which could be employed in clinical trials.^24 27–31^ Here, we focus on FAF, given it has been prioritised in clinical trial endpoints to date.^24 72 73^

In a ProgStar retrospective study of a subset of 224 eyes (mean age, 33.0±15.1 years), the total mean area of definitely decreased autofluorescence (DDAF) at the first visit was 2.6 mm^2^, and the mean progression of DDAF was 0.51 mm^2^/year.^72^ In a prospective study with 12 months of observation, the mean area of DDAF at baseline was 3.93 mm^2^, and the estimated progression of DDAF was 0.76 mm^2^/year.^73^ The rate of progression was dependent on the initial size of the lesion in both studies, as previously reported by other longitudinal studies.^12 74^

FAF imaging may serve as a monitoring tool for interventional clinical trials that aim to slow anatomical disease progression.^20 42 67^ Lesion size at baseline appears to be a strong predicting factor for lesion growth and can be partially accounted for by square root transformation.^12 20 42 67 75^

Studies using en face SD-OCT and OCT angiography (OCTA) have shown that the area of photoreceptor ellipsoid zone (EZ) loss was 1.6-fold greater than the area of RPE atrophy, which suggests that photoreceptor degeneration may precede RPE loss in STGD1.^76^ Moreover, OCTA showed that choriocapillaris vascular density was abnormal even beyond the areas of photoreceptor EZ and RPE loss, supporting a complex chorioretinal-RPE pathophysiology due to ABCA4 dysfunction.^76^ These findings may also be useful for developing end points in clinical trials.

## Therapeutic approaches

Although there are currently no proven cures for STGD1, there are multiple treatment avenues being investigated. In addition to retinal prosthesis,^77^ there are clinical trials of pharmacological agents, stem cell therapy and genetic therapies (see summary in table 1).^3 6–8 40–42 45^ Pharmacological therapies are arguably the most advanced and closest to potential approval as meaningful treatments.^6 7 42^

**Table 1:** Summary of therapeutic trials for Stargardt disease (STGD1; *ABCA4* retinopathy)

Mechanism	Treatment	Route	Phase	ClinicalTrials.gov identifier	Title	Summary results
Inhibition of vitamin A dimerisation	ALK-001	Oral	Phase 2 tong-term follow-up	NCT04239625	Open-Label Extension: Tolerability and Effects of ALK-001 on Stargardt Disease	Active study
Inhibition of vitamin A dimerisation	ALK-001	Oral	Phase 2	NCT02402660	Phase 2 Tolerability and Effects of ALK-001 on Stargardt Disease	Reduction in growth rate of atrophic lesions, no change in BCVA, no reports of night blindness or impaired dark adaptation
Inhibition of vitamin A dimerisation	ALK-001	Oral	Phase 1	NCT02230228	Phase 1 Safety Study of ALK-001 in Healthy Volunteers	
RBP4 Inhibition	STG-001	Oral	Phase 2	NCT04489511	Study of STG-001 in Subjects With Stargardt Disease	Reported AEs: 6 patients low dose: 1 dry eye, 1 subretinal fluid, 1 skin disorder; 4 patients high dose: 1 chromatopsia, 1 delayed dark adaptation, 2 night blindness, 1 visual impairment, 1 dry skin
RBP4 Inhibition	Tinlarebant	Oral	Phase 3	NCT05244304	Study to Evaluate the Safety and Efficacy of Tinlarebant in the Treatment of Stargardt Disease in Adolescent Subjects Lesion(s) in Adolescent Subjects With STGD1	Active study
RBP4 Inhibition	Tinlarebant	Oral	Phase 1 Phase 2	NCT05266014	Dose-finding Study Followed by 2 year Extension Study to Evaluate Safety and Tolerability of Tinlarebant in Adolescent Subjects With Stargardt Disease	Preliminary safety results: 9/13 patients delayed dark adaptation, 9/13 xanthopsia/chromatopsia, 1/13 night vision impairment. No clinically significant findings in relation to general health. 8/13 gain in BCVA, trend for preventing/slowing atrophy on FAF, 6/13 narrowing of EZ defect
RBP4 Inhibition	Vutrisiran	Subcutaneous	Phase 3	Not yet registered	THEIA-A: A Phase 3 Global, Randomised, Double-Masked, Placebo-Controlled Study to Evaluate the Clinical Outcomes, Efficacy and Safety of Vutrisiran in Patients with Stargardt Disease Type 1 (STGD1)	Upcoming trial
Inhibition of visual cycle (RPE65)	Emixustat	Oral	Phase 3	NCT03772665	Safety and Efficacy of Emixustat in Stargardt Disease	No meaningful differences between treatment groups regarding macular atrophy
Inhibition of visual cycle (RPE65)	Emixustat	Oral	Phase 2	NCT03033108	Pharmacodynamic Study of Emixustat Hydrochloride in Subjects With Macular Atrophy Secondary to Stargardt Disease	Dose-dependent suppression of rod b-wave amplitude recovery post photobleaching, confirming emixustat’s biological activity. AE: dark adaptation (11/23, 47.8%), erythropsia (5/23, 21.7%), vision blurred (4/23, 17.4%), photophobia (3/23, 13%), visual impairment (3/23, 13%), headache (2/23)
Inhibition of visual cycle	4-Methylpyrazole	Intravenous	Phase 1	NCT00346853	Phase 1 Pilot Study of 4-MP to Treat Stargardt Macular Dystrophy	No effect on dark adaptation in healthy probands, further studies suspended because substance doesn't seem to inhibit the visual cycle strong enough
Removal of lipofuscin	Soraprazan	Oral	Phase 2	EudraCT 2018-001496-20	A multinational, multi-centre, double-masked, placebo-controlled proof of concept trial to evaluate the safety and efficacy of oral soraprazan in Stargardt disease	Active study
Induce Autophagy	Metformin	Oral	Phase 1 Phase 2	NCT04545736	Oral Metformin for Treatment of ABCA4 Retinopathy	Active study
Inhibition of complement C5	Zimura	Intravitreal	Phase 2	NCT03364153	Zimura Compared with Sham in Patients With Autosomal Recessive Stargardt Disease (STGD1)	Active study
Supplements	Omega-3 Fatty Acids	Oral		NCT03297515	Therapeutic Potential of Omega-3 Fatty Acids Supplementation in Dry Macular Degeneration and Stargardt Disease	Increase of BCVA in the active group after 24 weeks, score of a questionnaire on perceived vision and subjective mood higher in the active group at week 24, CAVE: patient cohort Stargardt+dry AMD, results not shown separately
Supplements	Docosahexaenoic acid (DHA)	Oral		NCT00420602	DHA Supplementation in Patients With STGD3	No beneficial effect over 8 years, poor compliance
Supplements	DHA	Oral	Phase 1	NCT00060749	Effect of DHA Supplements on Macular Function in Patients With Stargardt Macular Dystrophy and Stargardt-like Macular Dystrophy	No effect on macular function
Supplements	Saffron	Oral	Phase 1 Phase 2	NCT01278277	Saffron Supplementation in Stargardt’s Disease	Short-term supplementation was well tolerated and had no detrimental effects on the electroretinographic responses of the central retina
Gene therapy (ABCA4)	SAR422459	Subretinal	Phase 1 Phase 2 Follow-up	NCT01736592	Phase I/II Follow-up Study of SAR422459 in Patients With Stargardt’s Macular Degeneration	Treatment was well tolerated. No clinically significant changes in visual function tests were found to be attributable to the treatment. Reduction of flecks in one eye. 1 case of ocular hypertension. 27% of treated eyes showed exacerbation of retinal pigment epithelium atrophy on FAF.
Gene therapy (ABCA4)	SAR422459	Subretinal	Phase 1 Phase 2	NCT01367444	Phase I/IIA Study of SAR422459 in Participants With Stargardt’s Macular Degeneration	Favourable safety profile
Optogenetics	vMCO-010	Intravitreal	Phase 2	NCT05417126	Safety and Effects of a Single Intravitreal Injection of vMCO-010 Optogenetic Therapy in Subjects With Stargardt Disease	Active study
Stem cells	hESC Derived RPE (MA09-hRPE)	Subretinal	Phase 2 Follow-up	NCT02941991	A Follow-up Study to Determine the Safety and Tolerability of Sub-retinal Transplantation of Human Embryonic Stem Cell Derived Retinal Pigmented Epithelial (hESC-RPE) Cells in Patients With Stargardt’s Macular Dystrophy (SMD)	Active study
Stem cells	hESC Derived RPE (MA09-hRPE)	Subretinal	Phase 1 Phase 2	NCT01345006	Sub-retinal Transplantation of hESC Derived RPE (MA09-hRPE) Cells in Patients With Stargardt’s Macular Dystrophy	No evidence of adverse proliferation, rejection, or serious ocular or systemic safety issues related to the transplanted tissue. 13/18 px (72%) had patches of increasing subretinal pigmentation. BCVA improved in ten eyes, improved or remained the same in seven eyes, and decreased by more than ten letters in one eye, no similar improvements in untreated FE. Vision-related quality-of-life measures increased 3–12 months after transplantation.
Stem cells	hESC Derived RPE (MA09-hRPE)	Subretinal	Follow-up	NCT02445612	Long Term Follow-up of Sub-retinal Transplantation of hESC Derived RPE Cells in Stargardt Macular Dystrophy Patients	Active study
Stem cells	hESC Derived RPE (MA09-hRPE)	Subretinal	Phase 1 Phase 2	NCT01469832	Safety and Tolerability of Sub-retinal Transplantation of hESC-RPE Cells in Patients With SMD	Focal areas of subretinal hyperpigmentation, no evidence of uncontrolled proliferation or inflammatory responses. No meaningful improvements in BCVA, no benefit in microperimetry at 12 months, one case of localised retinal thinning and reduced sensitivity in the area of hyperpigmentation. No significant change in participant-reported quality of life.
Stem cells	hESC Derived RPE (MA09-hRPE)	Subretinal	Phase 1	NCT01625559	A Phase I, Open-Label, Prospective Study to Determine the Safety and Tolerability of Sub-retinal Transplantation of hESC-RPE (MA09-hRPE) Cells in Patients With SMD	No serious AEs occurred throughout the 3 year period following the injection of hESC-RPE cells. The functional and anatomical results were favourable, compared with the natural course of SMD reported in the ProgStar study.
Stem cells	hESC Derived RPE	subretinal	Phase 1 Phase 2	NCT02749734	Clinical Study of Subretinal Transplantation of Human Embryo Stem Cell Derived Retinal Pigment Epitheliums in Treatment of Macular Degeneration Diseases	Active study
Stem cells	hESC Derived RPE	subretinal	Phase 1 Phase 2	NCT02903576	Stem Cell Therapy for Outer Retinal Degenerations (sub retinal injections vs hESC RPE seeded on a polymeric substrate implanted in the subretinal space)	Active study
Stem cells	Autologous bone marrow-isolated stem/progenitor cells	Intravitreal	Phase 1	NCT03772938	Stem Cells Therapy in Degenerative Diseases of the Retina	No results from STGD group to date
Stem cells	Autologous bone marrow derived stem cells (BMSC)	Retrobulbar, subtenon, intravitreal, intraocular, subretinal and intravenous		NCT03011541	Stem Cell Ophthalmology Treatment (SCOT) Study II	Active study
Stem cells	Autologous BMSC	Retrobulbar, subtenon, intravitreal, intraocular, subretinal and intravenous		NCT01920867	Stem Cell Ophthalmology Treatment Study	21/34 eyes (61.8%) improved, 8/34 eyes (23.5%) remained stable, and 5/34 eyes (14.7%) showed continued progression. The average central vision improvement following treatment was 17.96% and ranged up to 80.5%. Of 17 patients treated, 13/17px (76.5%) showed visual acuity improvement in one or both eyes, 3/17px (17.6%) showed no net loss, and 1px worsened as a consequence of disease progression; 94.1% of patients had improved vision or remained stable. There were no AEs.

AE, adverse event; AMD, age-related macular degeneration; BCVA, best-corrected VA; FAF, fundus autofluorescence ; VA, visual acuity.

### Pharmacological therapy

**Figure 3:**
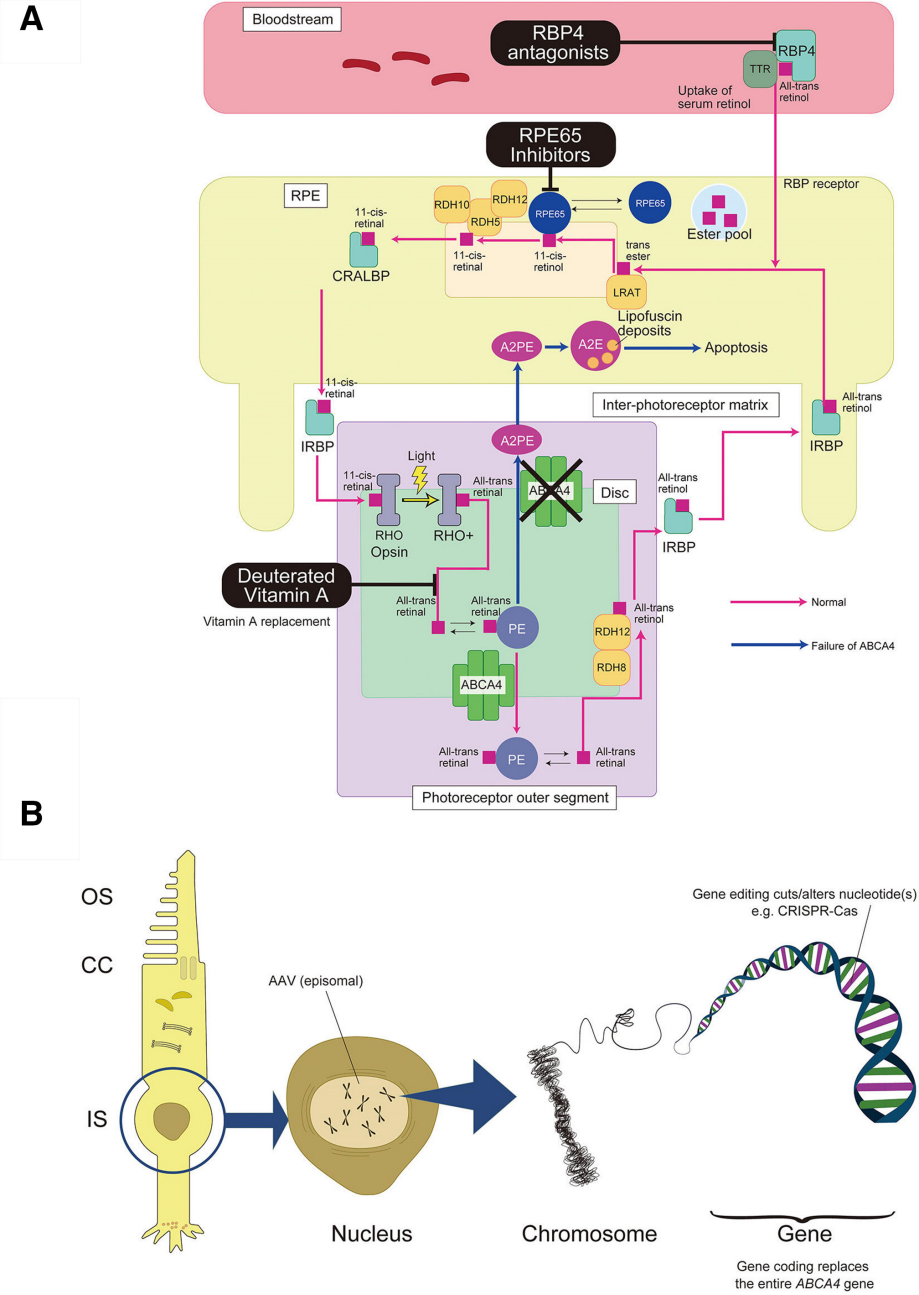
Current and future treatment agents for STGD1 A schematic showing (A) current pharmacological STGD1 treatment agents and (B) novel genetic therapies for STGD1. (A) Schematic showing the normal visual cycle (pink) and failure of transport due to ABCA4 dysfunction (blue). Agents (RPE65 inhibitors, deuterated Vitamin A, RBP4 antagonists) lower the formation of toxic products of the retinoid cycle by enzymatic inhibition, reducing delivery of vitamin A, or antagonising the retinoid binding protein 4 (RBP4). (B) Schematic showing non-integrating episomal and integrating nuclear gene therapies. The *ABCA4* gene is expressed in retinal photoreceptors and the transporter is localised at the rim of rod and cone photoreceptors at the outer segment (OS), which connects to the inner segment (IS) via connecting cilium (CC). To target disordered transport due to ABCA4 dysfunction, adeno-associated virus (AAV) therapies deliver the large 6.4 kb *ABCA4* gene (>4.7 kb AAV cargo limit) to the nucleus by splicing together fragments of the *ABCA4* gene, wherein the transgene remains in an episomal state. Gene editing therapies cut or alter single nucleotide(s) within the *ABCA4* gene via techniques such as CRISPR-Cas, which targets specific variants. Gene coding replaces the entire *ABCA4* gene via an engineered transposase, enabling its application to all variants, including exonic and intronic nucleotide variants, as well as structural variants.

Several pharmacological agents have been specifically developed that target different aspects of the retinoid cycle and are potentially beneficial in slowing or preventing progression in STGD (figure 3A), with some studies also reporting improvements in retinal and/or visual function (table 1).^3 6 8 42^

The aims of these agents are either (1) lowering the formation of toxic products of the retinoid cycle by reducing delivery of vitamin A or inhibition of various enzymes participating in the cycle, including drugs such as emixustat,^78–80^ ALK-001, LBS-008, STG-001, fenretinide and A1120; or (2) directly targeting toxic metabolites such as A2E or pathways activated by these metabolites (eg, the complement cascade), including soraprazan and Avacincaptad pegol.

These drugs aim to impede formation of A2E and lipofuscin by either slowing the rate of vitamin A dimerisation (ALK-001),^81 82^ enhancing lipofuscin removal (soraprazan), imposing competitive inhibitory mechanisms on the retinal binding protein-4 (LBS-008 (tinlarebant), STG-001, fenretinide, vutrisiran and A1120), or modulating the activity of RPE65 (emixustat).^78^

Many of these drugs have been or are currently in phase 1/2 or 3 trials (LBS-008: NCT03735810, emixustat: NCT03772665 and NCT03033108, ALK-001: NCT02402660) (table 1). Avacincaptad pegol, a complement C5 inhibitor, is also being investigated in a phase 2 trial (NCT03364153). Additional pharmacotherapeutic agents directly or indirectly targeting the visual cycle have been developed, including the complement-mediated response to accumulated by-products of the visual cycle.^83^

### Cellular therapies

For the management of advanced disease, cell replacement strategies offer potential benefit. A phase 1/2 clinical trial (NCT01469832) of human embryonic stem cell (hESC)-derived RPE cells for treating severe advanced STGD1 has been completed.^43 84^ Findings from the UK site of this trial identified subretinal hyperpigmentation consistent with the survival of viable transplanted hESC-derived RPE cells.^84^ Borderline improvements in VA were noted in 4 of 12 patients; however, microperimetry did not demonstrate evidence of functional benefit at 12 months.^84^ A phase 1 clinical trial testing the long-term safety and tolerability of hESC-derived RPE (NCT01625559) showed no adverse events, with favourable results.^85^ Further trials are anticipated, including evaluation of combined RPE and photoreceptor transplants, which are either derived from hESCs or induced pluripotent stem cells (iPSC).

Trials involving autologous bone marrow-derived stem cells (BMSC; NCT01920867, NCT03011541, NCT03772938) are at various stages of completion. One study (NCT01920867) showed improvement in 61.8% of the eyes treated, with 76.9% of patients exhibiting VA improvement. Other studies involving BMSC treatment (NCT03772938, NCT03011541) are still active with no results yet reported.

### Genetic therapies

Gene replacement therapy has been increasingly applied to photoreceptor diseases, aiming to slow or prevent further degeneration and/or improve function in early to intermediate stage disease.^7 40–42 86^ Preclinical studies in gene replacement showing phenotypic improvement in abca4^−/−^ mice have subsequently encouraged the development of human gene therapy trials.^87 88^ Adeno-associated virus (AAV) vectors have been the leading choice for gene delivery in human gene therapy; however, the AAV capsids exhibit limited cargo capacity. The *ABCA4* gene is far larger than the current AAV vector capacity.^87^ Considering the larger cargo capacity of lentiviruses, subretinal injection of a lentiviral vector delivering *ABCA4* (SAR422459) was developed. The StarGen phase I/II trial for this therapy (NCT01367444) was terminated early, with a longer-term follow-up study ongoing (NCT01736592).^89^ Although there were no safety concerns in either of these trials, there was no evidence of visual improvement.^46 90^

Optogenetics represents a genetic therapy for advanced disease, where residual non-photoreceptor cells are made light sensitive by using AAV to deliver often an opsin-related photopigment.^91^ This approach is being explored in a Phase II clinical trial in STGD1, with AAV2 carrying a multicharacteristic opsin gene expression cassette (NCT05417126).^92^

### Future treatment options

In addition to treatments currently undergoing clinical trials, there are several therapeutic approaches on the horizon for STGD1.^93^ Anti-sense oligonucleotide (AON) treatments have exhibited great potential for the personalised treatment of patients that carry one of the *ABCA4* splice variants.^55 57^ Phase I/II clinical trials for the use of an AON-based therapeutic intravitreal injection to treat Leber congenital amaurosis (NCT03140969, NCT03913130), retinitis pigmentosa and Usher syndrome (NCT05085964) were conducted, but two of these studies (NCT03913130, NCT05085964) were terminated early for reasons unrelated to safety. Research involving the application of AON-based therapy has recently shown promise at preventing further deterioration in Stargardt disease models.^94^

Other therapeutic methods being explored include gene therapy systems that use alternative delivery vectors. As mentioned above, there are fundamental limitations to using AAVs in STGD1, principally cargo capacity, as well as concerns about immune reactions to the viral vector itself.^95 96^ Thus, future treatment methodologies that employ non-viral vectors—such as cationic lipids and lipid nanoparticles (LNPs)—would be potentially safer than viral vector options with respect to the absence of immunogenic viral proteins. LNPs have also shown robust capability to condense and deliver various nucleic acid molecules up to several million nucleotides,^97^ while concurrently protecting the DNA/RNA cargo from unknown chromosomal position effects.^98 99^ However, the level of expression and transfection efficiency for non-viral vectors is typically much lower than viral vectors. Recent strategies such as PEGylation of LNPs or use of a pH-sensitive amino lipid have been shown to markedly enhance efficiency and targeting of ocular delivery.^100 101^ Other approaches being developed include either using dual or triple AAV vectors to deliver full length *ABCA4* to the nucleus by splicing together fragments of the cDNA.^102–104^

Novel CRISPR-based molecular tools have also emerged as a therapeutic option for STGD1 (figure 3B).^105^ Recently, gene editing via CRISPR/Cas9 has been employed to correct pathogenic variants of *ABCA4* in human iPSCs (hiPSC) for STGD1 patients.^106^ However, there are potential safety concerns associated with gene editing methods being developed for STGD1, namely the introduction of double-stranded breaks (DSBs) in the genome during editing. Gene editing systems such as CRISPR/Cas9 create DSBs, which run the risk of triggering error-prone endogenous DNA repair mechanisms that could otherwise cause unwanted effects.^107 108^ However, systems that exploit transposon-based mutagenesis—such as fish-derived Sleeping Beauty^109 110^ and insect-derived PiggyBac^111^—may circumvent this issue. A potential drawback for such methods is that the DNA recognition sequence may be found throughout the human genome and thus the gene would not be targeted to a specific site. Thus, an ideal transposon-based system would be one that is mammal-derived and absent of immunogenic effects, with the capability to insert genetic material of unlimited size at a site-specific genomic target.

By combining features of the above systems, there is potential for developing much needed novel gene therapies that can transport a larger size DNA cargo (and avoid the introduction of DSBs) for STGD1 and other common IRDs such as *USH2A*-associated retinitis pigmentosa and Usher syndrome. For example, a novel DNA integrating platform developed by SalioGen Therapeutics—Gene Coding—combines many of the above-mentioned features (figure 3B). The technology uses a tissue-specific and cell-specific nanoparticle (NP) to co-encapsulate mRNA encoding a synthetic bioengineered, mammalian transposase and a DNA element containing the gene of interest for a specific disease target. Notably, the DNA element can contain large genetic cargos, such as *ABCA4,* or a combination of several genetic factors, since the NP does not have size limitations.

Several potential benefits of this type of technology are currently under investigation. First, NPs that target specific cell types such as photoreceptors and RPE are being developed.^112^ Second, the NP capability to deliver large gene cargos is being exploited to deliver single or multiple genetic components and regulatory elements to control gene expression. Third, the non-viral nature of the DNA integration system may decrease the immunogenicity seen with viral delivery systems. Finally, in contrast to AAV therapies and non-viral gene editing technologies and gene therapies, the transposase avoids unwanted genomic effects by avoiding DSBs^113^ while integrating therapeutic genes at polynucleotide sites in the genome. All of these potential attributes may be important in treating degenerative retinal disorders such as STGD1, which is caused by multiple pathogenic variants in large genes.

## Conclusions

STGD1 is one of the most common IRDs, presenting in childhood, early adulthood and in later life. This *ABCA4*-associated retinopathy is highly heterogeneous both clinically and genetically. The deep clinical and genetic characterisation that has been undertaken over the last 15 years has improved understanding of underlying disease mechanisms, natural history and outcome metrics, allowing multiple therapeutic trials to be conducted. Further trials are anticipated, including pharmacological in the immediate term, with innovations towards the development of novel gene therapy approaches on the horizon.

## Data Availability

No data are available.

## References

[1] Michaelides M , Hunt DM , Moore AT . The Genetics of inherited macular dystrophies. J Med Genet 2003;40:641–50. 10.1136/jmg.40.9.641 12960208 PMC1735576

[2] Michaelides M , Hardcastle AJ , Hunt DM , et al . Progressive cone and cone-rod dystrophies: phenotypes and underlying molecular genetic basis. Surv Ophthalmol 2006;51:232–58. 10.1016/j.survophthal.2006.02.007 16644365

[3] Tanna P , Strauss RW , Fujinami K , et al . Stargardt disease: clinical features, molecular genetics, animal models and therapeutic options. Br J Ophthalmol 2017;101:25–30. 10.1136/bjophthalmol-2016-308823 27491360 PMC5256119

[4] Gill JS , Georgiou M , Kalitzeos A , et al . Progressive cone and cone-rod dystrophies: clinical features, molecular genetics and prospects for therapy. Br J Ophthalmol 2019;103:711–20. 10.1136/bjophthalmol-2018-313278 30679166 PMC6709772

[5] Liu X , Fujinami YY , Yang L , et al . Stargardt disease in Asian population. In: Advances in Vision Research. Singapore: Springer, 2019: 279–95. 10.1007/978-981-13-0884-0

[6] Cremers FPM , Lee W , Collin RWJ , et al . Clinical spectrum, genetic complexity and therapeutic approaches for retinal disease caused by Abca4 mutations. Prog Retin Eye Res 2020;79:100861. 10.1016/j.preteyeres.2020.100861 32278709 PMC7544654

[7] Rahman N , Georgiou M , Khan KN , et al . Macular dystrophies: clinical and imaging features, molecular genetics and therapeutic options. Br J Ophthalmol 2020;104:451–60. 10.1136/bjophthalmol-2019-315086 31704701 PMC7147237

[8] Fujinami KF-Y , Yang L , Liu X , et al . East Asia inherited retinal disease society (Eairds) study group. Stargardt macular dystrophy. In: Yu HG , ed. Inherited Retinal Disease 2022. n.d.: 151–68. 10.1007/978-981-16-7337-5

[9] Stargardt K . Über Familiäre, progressive degeneration in der maculagegend des auges. Graefes Arhiv Für Ophthalmologie 1909;71:534–50. 10.1007/BF01961301

[10] Michaelides M , Chen LL , Brantley MA , et al . Abca4 mutations and discordant Abca4 Alleles in patients and siblings with bull’S-eye maculopathy. British Journal of Ophthalmology 2007;91:1650–5. 10.1136/bjo.2007.118356 18024811 PMC2095527

[11] Fujinami K , Lois N , Davidson AE , et al . A longitudinal study of stargardt disease: clinical and electrophysiologic assessment, progression, and genotype correlations. American Journal of Ophthalmology 2013;155:1075–1088. 10.1016/j.ajo.2013.01.018 23499370

[12] Fujinami K , Lois N , Mukherjee R , et al . A longitudinal study of stargardt disease: quantitative assessment of fundus autofluorescence, progression, and genotype correlations. Invest Ophthalmol Vis Sci 2013;54:8181. 10.1167/iovs.13-12104 24265018

[13] Fujinami K , Sergouniotis PI , Davidson AE , et al . The clinical effect of homozygous Abca4 Alleles in 18 patients. Ophthalmology 2013;120:2324–31. 10.1016/j.ophtha.2013.04.016 23769331

[14] Fujinami K , Sergouniotis PI , Davidson AE , et al . Clinical and molecular analysis of stargardt disease with preserved foveal structure and function. American Journal of Ophthalmology 2013;156:487–501. 10.1016/j.ajo.2013.05.003 23953153

[15] Fujinami K , Singh R , Carroll J , et al . Fine central macular dots associated with childhood-onset stargardt disease. Acta Ophthalmol 2014;92:e157–9. 10.1111/aos.12259 24020726

[16] Fujinami K , Zernant J , Chana RK , et al . Clinical and molecular characteristics of childhood-onset stargardt disease. Ophthalmology 2015;122:326–34. 10.1016/j.ophtha.2014.08.012 25312043 PMC4459618

[17] Khan KN , Kasilian M , Mahroo OAR , et al . Early patterns of macular degeneration in Abca4-associated retinopathy. Ophthalmology 2018;125:735–46. 10.1016/j.ophtha.2017.11.020 29310964 PMC5917070

[18] Tanna P , Georgiou M , Aboshiha J , et al . Cross-sectional and longitudinal assessment of retinal sensitivity in patients with childhood-onset stargardt disease. Transl Vis Sci Technol 2018;7:10. 10.1167/tvst.7.6.10 PMC6262645 30510854

[19] Tanna P , Georgiou M , Strauss RW , et al . Cross-sectional and longitudinal assessment of the ellipsoid zone in childhood-onset stargardt disease. Transl Vis Sci Technol 2019;8:1. 10.1167/tvst.8.2.1 PMC6397016 30834176

[20] Georgiou M , Kane T , Tanna P , et al . Prospective cohort study of childhood-onset stargardt disease: fundus autofluorescence imaging, progression, comparison with adult-onset disease, and disease symmetry. Am J Ophthalmol 2020;211:159–75. 10.1016/j.ajo.2019.11.008 31812472 PMC7082771

[21] Fishman GA , Stone EM , Grover S , et al . Variation of clinical expression in patients with stargardt dystrophy and sequence variations in the ABCR gene. Arch Ophthalmol 1999;117:504–10. 10.1001/archopht.117.4.504 10206579

[22] Lois N , Holder GE , Bunce C , et al . Phenotypic subtypes of stargardt macular dystrophy-fundus flavimaculatus. Arch Ophthalmol 2001;119:359–69. 10.1001/archopht.119.3.359 11231769

[23] Fujinami K , Zernant J , Chana RK , et al . Abca4 gene screening by next-generation sequencing in a British cohort. Invest Ophthalmol Vis Sci 2013;54:6662–74. 10.1167/iovs.13-12570 23982839 PMC3796939

[24] Strauss RW , Ho A , Muñoz B , et al . The natural history of the progression of atrophy secondary to stargardt disease (Progstar) studies: design and baseline characteristics: progstar report no. 1. Ophthalmology 2016;123:817–28. 10.1016/j.ophtha.2015.12.009 26786511

[25] Fakin A , Robson AG , Fujinami K , et al . Phenotype and progression of retinal degeneration associated with nullizigosity of Abca4. Invest Ophthalmol Vis Sci 2016;57:4668–78. 10.1167/iovs.16-19829 27583828

[26] Fakin A , Robson AG , Chiang JP-W , et al . The effect on retinal structure and function of 15 specific Abca4 mutations: a detailed examination of 82 hemizygous patients. Invest Ophthalmol Vis Sci 2016;57:5963–73. 10.1167/iovs.16-20446 27820952

[27] Kong X , Fujinami K , Strauss RW , et al . Visual acuity change over 24 months and its association with foveal phenotype and genotype in individuals with stargardt disease [ProgStar Study Report no.10]. JAMA Ophthalmol 2018;136:920–8. 10.1001/jamaophthalmol.2018.2198 29902293 PMC6142940

[28] Fujinami K , Strauss RW , Chiang J (Pei-W , et al . Detailed genetic characteristics of an international large cohort of patients with stargardt disease [ProgStar study report 8]. Br J Ophthalmol 2019;103:390–7. 10.1136/bjophthalmol-2018-312064 29925512 PMC6579578

[29] Schönbach EM , Strauss RW , Cattaneo M , et al . Longitudinal changes of fixation stability and location within 24 months in stargardt disease: progstar report no. 16. Am J Ophthalmol 2022;233:78–89. 10.1016/j.ajo.2021.07.013 34298008

[30] Schönbach EM , Janeschitz-Kriegl L , Strauss RW , et al . The progression of stargardt disease using volumetric hill of vision analyses over 24 months [ProgStar Report No.15]. American Journal of Ophthalmology 2021;230:123–33. 10.1016/j.ajo.2021.04.015 33951446

[31] Schönbach EM , Strauss RW , Ibrahim MA , et al . Faster sensitivity loss around dense scotomas than for overall macular sensitivity in stargardt disease [ProgStar Report no.14]. American Journal of Ophthalmology 2020;216:219–25. 10.1016/j.ajo.2020.03.020 32222369

[32] Liu X , Meng X , Yang L , et al . Clinical and genetic characteristics of Stargardt disease in a large Western China cohort: report 1 [Report 1]. Am J Med Genet C Semin Med Genet 2020;184:694–707. 10.1002/ajmg.c.31838 32845068

[33] Hanany M , Rivolta C , Sharon D . Worldwide carrier frequency and genetic prevalence of Autosomal Recessive inherited retinal diseases. Proc Natl Acad Sci U S A 2020;117:2710–6. 10.1073/pnas.1913179117 31964843 PMC7007541

[34] Galvin O , Chi G , Brady L , et al . The impact of inherited retinal diseases in the Republic of Ireland (ROI) and the United Kingdom (UK) from a cost-of-illness perspective. Clin Ophthalmol 2020;14:707–19. 10.2147/OPTH.S241928 32184557 PMC7062501

[35] Wittenborn JS , Zhang X , Feagan CW , et al . The economic burden of vision loss and eye disorders among the United States population younger than 40 years. Ophthalmology 2013;120:1728–35. 10.1016/j.ophtha.2013.01.068 23631946 PMC5304763

[36] Aziz K , Swenor BK , Canner JK , et al . The direct healthcare cost of stargardt disease: a claims-based analysis. Ophthalmic Epidemiol 2021;28:533–9. 10.1080/09286586.2021.1883675 33615979 PMC11207193

[37] Allikmets R , Singh N , Sun H , et al . A Photoreceptor cell-specific ATP-binding transporter gene (ABCR) is Mutated in recessive stargardt macular dystrophy. Nat Genet 1997;15:236–46. 10.1038/ng0397-236 9054934

[38] Burke TR , Tsang SH . Allelic and phenotypic heterogeneity in Abca4 mutations. Ophthalmic Genet 2011;32:165–74. 10.3109/13816810.2011.565397 21510770 PMC3155666

[39] Lorenz B , Preising MN . Age matters—thoughts on a grading system for Abca4 mutations. Graefe’s Arch Clin Exp Ophthalmol 2005;243:87–9. 10.1007/s00417-004-1078-5 15614538

[40] Smith J , Ward D , Michaelides M , et al . New and emerging technologies for the treatment of inherited retinal diseases: a horizon scanning review. Eye 2015;29:1131–40. 10.1038/eye.2015.115 26113499 PMC4565944

[41] Scholl HPN , Strauss RW , Singh MS , et al . Emerging therapies for inherited retinal degeneration. Sci Transl Med 2016;8:368.:368rv6. 10.1126/scitranslmed.aaf2838 27928030

[42] Georgiou M , Fujinami K , Michaelides M . Inherited retinal diseases: therapeutics, clinical trials and end points-A review. Clin Exp Ophthalmol 2021;49:270–88. 10.1111/ceo.13917 33686777

[43] Schwartz SD , Regillo CD , Lam BL , et al . Human embryonic stem cell-derived retinal pigment epithelium in patients with age-related macular degeneration and stargardt’s macular dystrophy: follow-up of two open-label phase 1/2 studies. The Lancet 2015;385:509–16. 10.1016/S0140-6736(14)61376-3 25458728

[44] Li S-Y , Liu Y , Wang L , et al . A phase I clinical trial of human embryonic stem cell-derived retinal pigment epithelial cells for early-stage stargardt macular degeneration: 5-years' follow-up. Cell Prolif 2021;54:e13100. 10.1111/cpr.13100 34347352 PMC8450131

[45] Ku CA , Yang P . Stargardt disease: gene therapy strategies for Abca4. Int Ophthalmol Clin 2021;61:157–65. 10.1097/IIO.0000000000000375 34584053

[46] Parker MA , Erker LR , Audo I , et al . Three-year safety results of Sar422459 (EIAV-Abca4) gene therapy in patients with Abca4-associated stargardt disease: an open-label dose-escalation phase I/IIa clinical trial, cohorts 1-5. Am J Ophthalmol 2022;240:285–301. 10.1016/j.ajo.2022.02.013 35248547 PMC9308722

[47] Levi SR , Ryu J , Liu P-K , et al . Precision medicine trials in retinal degenerations. Annu Rev Vis Sci 2021;7:851–65. 10.1146/annurev-vision-100419-111701 34524878

[48] Tsybovsky Y , Molday RS , Palczewski K . The ATP-binding cassette transporter Abca4: structural and functional properties and role in retinal disease. Adv Exp Med Biol 2010;703:105–25. 10.1007/978-1-4419-5635-4_8 20711710 PMC2930353

[49] Sun H , Nathans J . ABCR: rod photoreceptor-specific ABC transporter responsible for stargardt disease. Methods Enzymol 2000;315:879–97. 10.1016/s0076-6879(00)15888-4 10736747

[50] Cideciyan AV , Aleman TS , Swider M , et al . Mutations in Abca4 result in accumulation of lipofuscin before slowing of the retinoid cycle: a reappraisal of the human disease sequence. Hum Mol Genet 2004;13:525–34. 10.1093/hmg/ddh048 14709597

[51] Charbel Issa P , Barnard AR , Singh MS , et al . Fundus autofluorescence in the Abca4(-/-) mouse model of stargardt disease--correlation with accumulation of A2E, retinal function, and histology. Invest Ophthalmol Vis Sci 2013;54:5602–12. 10.1167/iovs.13-11688 23761084 PMC3747716

[52] Radu RA , Mata NL , Bagla A , et al . Light exposure stimulates formation of A2E oxiranes in a mouse model of stargardt’s macular degeneration. Proc Natl Acad Sci U S A 2004;101:5928–33. 10.1073/pnas.0308302101 15067110 PMC395900

[53] Sparrow JR , Boulton M . RPE lipofuscin and its role in retinal pathobiology. Experimental Eye Research 2005;80:595–606. 10.1016/j.exer.2005.01.007 15862166

[54] Lenis TL , Hu J , Ng SY , et al . Expression of Abca4 in the retinal pigment epithelium and its implications for stargardt macular degeneration. Proc Natl Acad Sci U S A 2018;115:E11120–7. 10.1073/pnas.1802519115 30397118 PMC6255167

[55] Sangermano R , Garanto A , Khan M , et al . Deep-Intronic Abca4 variants explain missing heritability in stargardt disease and allow correction of splice defects by antisense oligonucleotides. Genetics in Medicine 2019;21:1751–60. 10.1038/s41436-018-0414-9 30643219 PMC6752325

[56] Al-Khuzaei S , Broadgate S , Foster CR , et al . An overview of the genetics of Abca4 retinopathies, an evolving story. Genes (Basel) 2021;12:1241. 10.3390/genes12081241 34440414 PMC8392661

[57] Tomkiewicz TZ , Suárez-Herrera N , Cremers FPM , et al . Antisense oligonucleotide-based rescue of aberrant splicing defects caused by 15 pathogenic variants in Abca4 Int J Mol Sci 2021;22:4621. 10.3390/ijms22094621 33924840 PMC8124656

[58] Heath Jeffery RC , Thompson JA , Lo J , et al . n.d. Genotype-specific lesion growth rates in stargardt disease. Genes;12:1981. 10.3390/genes12121981 PMC8701386 34946930

[59] Zernant J , Lee W , Collison FT , et al . Frequent hypomorphic alleles account for a significant fraction of Abca4 disease and distinguish it from age-related macular degeneration. J Med Genet 2017;54:404–12. 10.1136/jmedgenet-2017-104540 28446513 PMC5786429

[60] Mena MD , Moresco AA , Vidal SH , et al . Clinical and genetic spectrum of stargardt disease in Argentinean patients. Front Genet 2021;12:646058. 10.3389/fgene.2021.646058 33841504 PMC8033171

[61] Joo K , Seong MW , Park KH , et al . Genotypic profile and phenotype correlations of Abca4-associated retinopathy in Koreans. Mol Vis 2019;25:679–90. 31814693 PMC6857773

[62] Lee W , Zernant J , Su PY , et al . A genotype-phenotype correlation matrix for Abca4 disease based on long-term prognostic outcomes. JCI Insight 2022;7:e156154. 10.1172/jci.insight.156154 34874912 PMC8855796

[63] Sangermano R , Khan M , Cornelis SS , et al . Abca4 midigenes reveal the full splice spectrum of all reported noncanonical splice site variants in stargardt disease. Genome Res 2018;28:100–10. 10.1101/gr.226621.117 29162642 PMC5749174

[64] Huang D , Thompson JA , Chen S-C , et al . Characterising splicing defects of Abca4 variants within Exons 13-50 in patient-derived fibroblasts. Exp Eye Res 2022;225:109276. 10.1016/j.exer.2022.109276 36209838

[65] Weng J , Mata NL , Azarian SM , et al . Insights into the function of rim protein in photoreceptors and etiology of stargardt’s disease from the phenotype in Abcr knockout mice. Cell 1999;98:13–23. 10.1016/S0092-8674(00)80602-9 10412977

[66] Georgiou M , Kalitzeos A , Patterson EJ , et al . Adaptive optics imaging of inherited retinal diseases. Br J Ophthalmol 2018;102:1028–35. 10.1136/bjophthalmol-2017-311328 29141905 PMC6059037

[67] Georgiou M , Fujinami K , Michaelides M . Retinal imaging in inherited retinal diseases. Ann Eye Sci 2020;5:25. 10.21037/aes-20-81 33928237 PMC8081382

[68] Bach M , Brigell MG , Hawlina M , et al . ISCEV standard for clinical pattern electroretinography (PERG): 2012 update. Doc Ophthalmol 2013;126:1–7. 10.1007/s10633-012-9353-y 23073702

[69] Hoffmann MB , Bach M , Kondo M , et al . ISCEV standard for clinical multifocal electroretinography (mfERG) (2021 update). Doc Ophthalmol 2021;142:5–16. 10.1007/s10633-020-09812-w 33492495 PMC7906932

[70] Robson AG , Frishman LJ , Grigg J , et al . ISCEV standard for full-field clinical electroretinography (2022 update). Doc Ophthalmol 2022;144:165–77. 10.1007/s10633-022-09872-0 35511377 PMC9192408

[71] Fujinami K , Akahori M , Fukui M , et al . Stargardt disease with preserved central vision: identification of a putative novel Mutation in ATP-binding cassette transporter gene. Acta Ophthalmol 2011;89:e297–8. 10.1111/j.1755-3768.2009.01848.x 20163366

[72] Strauss RW , Muñoz B , Ho A , et al . Progression of stargardt disease as determined by fundus autofluorescence in the retrospective progression of Stargardt disease study [ProgStar Report no.9]. JAMA Ophthalmol 2017;135:1232. 10.1001/jamaophthalmol.2017.4152 29049437 PMC5710470

[73] Strauss RW , Kong X , Ho A , et al . Progression of stargardt disease as determined by fundus autofluorescence over a 12-month period [ProgStar Report no.11]. JAMA Ophthalmol 2019;137:1134–45. 10.1001/jamaophthalmol.2019.2885 31369039 PMC6681653

[74] McBain VA , Townend J , Lois N . Progression of retinal pigment epithelial atrophy in stargardt disease. American Journal of Ophthalmology 2012;154:146–54. 10.1016/j.ajo.2012.01.019 22464366

[75] Heath Jeffery RC , Chen FK . Stargardt disease: multimodal imaging: a review. Clin Exp Ophthalmol 2021;49:498–515. 10.1111/ceo.13947 34013643 PMC8366508

[76] Alabduljalil T , Patel RC , Alqahtani AA , et al . Correlation of outer retinal degeneration and choriocapillaris loss in stargardt disease using en face optical coherence tomography and optical coherence tomography angiography. Am J Ophthalmol 2019;202:79–90. 10.1016/j.ajo.2019.02.007 30771335 PMC6548611

[77] Bloch E , Luo Y , da Cruz L . Advances in retinal prosthesis systems. Ther Adv Ophthalmol 2019;11:2515841418817501. 10.1177/2515841418817501 30729233 PMC6350159

[78] Kubota R , Boman NL , David R , et al . Safety and effect on rod function of ACU-4429, a novel small-molecule visual cycle modulator. Retina 2012;32:183–8. 10.1097/IAE.0b013e318217369e 21519291

[79] Kubota R , Al-Fayoumi S , Mallikaarjun S , et al . Phase 1, dose-ranging study of emixustat hydrochloride (ACU-4429), a novel visual cycle modulator, in healthy volunteers. Retina 2014;34:603–9. 10.1097/01.iae.0000434565.80060.f8 24056528

[80] Dugel PU , Novack RL , Csaky KG , et al . Phase II, randomized, placebo-controlled, 90-day study of emixustat hydrochloride in geographic atrophy associated with dry age-related macular degeneration. Retina 2015;35:1173–83. 10.1097/IAE.0000000000000606 25932553 PMC4452434

[81] Kaufman Y , Ma L , Washington I . Deuterium enrichment of vitamin A at the C20 position SLOWS the formation of detrimental vitamin A dimers in wild-type rodents. Journal of Biological Chemistry 2011;286:7958–65. 10.1074/jbc.M110.178640 21075840 PMC3048682

[82] Charbel Issa P , Barnard AR , Herrmann P , et al . Rescue of the stargardt phenotype in Abca4 knockout mice through inhibition of vitamin A dimerization. Proc Natl Acad Sci U S A 2015;112:8415–20. 10.1073/pnas.1506960112 26106163 PMC4500285

[83] Lu LJ , Liu J , Adelman RA . Novel therapeutics for stargardt disease. Graefes Arch Clin Exp Ophthalmol 2017;255:1057–62. 10.1007/s00417-017-3619-8 28285324

[84] Mehat MS , Sundaram V , Ripamonti C , et al . Transplantation of human embryonic stem cell-derived retinal pigment epithelial cells in macular degeneration. Ophthalmology 2018;125:1765–75. 10.1016/j.ophtha.2018.04.037 29884405 PMC6195794

[85] Sung Y , Lee MJ , Choi J , et al . Long-term safety and tolerability of subretinal transplantation of embryonic stem cell-derived retinal pigment epithelium in Asian stargardt disease patients. Br J Ophthalmol 2021;105:829–37. 10.1136/bjophthalmol-2020-316225 32727729

[86] Vázquez-Domínguez I , Garanto A , Collin RWJ . Molecular therapies for inherited retinal diseases-current standing. Genes (Basel) 2019;10:654. 10.3390/genes10090654 31466352 PMC6770110

[87] Allocca M , Doria M , Petrillo M , et al . Serotype-dependent packaging of large genes in adeno-associated viral vectors results in effective gene delivery in mice. J Clin Invest 2008;118:1955–64. 10.1172/JCI34316 18414684 PMC2298836

[88] Han Z , Conley SM , Makkia RS , et al . DNA nanoparticle-mediated Abca4 delivery rescues stargardt dystrophy in mice. J Clin Invest 2012;122:3221–6.:64833. 10.1172/JCI64833 22886305 PMC3428101

[89] Parker MA , Choi D , Erker LR , et al . Test-retest variability of functional and structural parameters in patients with stargardt disease participating in the Sar422459 gene therapy trial. Transl Vis Sci Technol 2016;5:10. 10.1167/tvst.5.5.10 PMC5054761 27730010

[90] Dalkara D , Goureau O , Marazova K , et al . Let there be light: gene and cell therapy for blindness. Hum Gene Ther 2016;27:134–47. 10.1089/hum.2015.147 26751519 PMC4779297

[91] Hulliger EC , Hostettler SM , Kleinlogel S . Empowering retinal gene therapy with a specific promoter for human rod and cone ON-bipolar cells. Mol Ther Methods Clin Dev 2020;17:505–19. 10.1016/j.omtm.2020.03.003 32258214 PMC7114634

[92] Wright W , Gajjeraman S , Batabyal S , et al . Restoring vision in mice with retinal degeneration using multicharacteristic Opsin. Neurophotonics 2017;4:049801. 10.1117/1.NPh.4.4.049801 28948190 PMC5603575

[93] Girach A , Audo I , Birch DG , et al . RNA-based therapies in inherited retinal diseases. Ther Adv Ophthalmol 2022;14:25158414221134602. 10.1177/25158414221134602 36388727 PMC9643766

[94] Kaltak M , de Bruijn P , Piccolo D , et al . Antisense oligonucleotide therapy corrects splicing in the common stargardt disease type 1-causing variant Abca4 C.5461-10T>C. Mol Ther Nucleic Acids 2023;31:674–88. 10.1016/j.omtn.2023.02.020 36910710 PMC9999166

[95] Bucher K , Rodríguez-Bocanegra E , Dauletbekov D , et al . Immune responses to retinal gene therapy using adeno-associated viral vectors - implications for treatment success and safety. Prog Retin Eye Res 2021;83:100915. 10.1016/j.preteyeres.2020.100915 33069860

[96] Chandler LC , McClements ME , Yusuf IH , et al . Characterizing the cellular immune response to subretinal AAV gene therapy in the murine retina. Mol Ther Methods Clin Dev 2021;22:52–65. 10.1016/j.omtm.2021.05.011 34485594 PMC8390455

[97] Zhao Y , Huang L . Lipid nanoparticles for gene delivery. Adv Genet 2014;88:13–36. 10.1016/B978-0-12-800148-6.00002-X 25409602 PMC5006671

[98] Kulkarni JA , Cullis PR , van der Meel R . Lipid nanoparticles enabling gene therapies: from concepts to clinical utility. Nucleic Acid Ther 2018;28:146–57. 10.1089/nat.2018.0721 29683383

[99] Li L , Hu S , Chen X . Non-viral delivery systems for CRISPR/Cas9-based genome editing: challenges and opportunities. Biomaterials 2018;171:207–18. 10.1016/j.biomaterials.2018.04.031 29704747 PMC5944364

[100] Ryals RC , Patel S , Acosta C , et al . The effects of pegylation on LNP based mRNA delivery to the eye. PLoS One 2020;15:e0241006. 10.1371/journal.pone.0241006 33119640 PMC7595320

[101] Sun D , Sun W , Gao S-Q , et al . Effective gene therapy of stargardt disease with PEG-ECO/Pgrk1-Abca4-S/MAR nanoparticles. Molecular Therapy - Nucleic Acids 2022;29:823–35. 10.1016/j.omtn.2022.08.026 36159595 PMC9463552

[102] McClements ME , Barnard AR , Singh MS , et al . An AAV dual vector strategy ameliorates the stargardt phenotype in adult Abca4(-/-) mice. Hum Gene Ther 2019;30:590–600. 30381971 10.1089/hum.2018.156 PMC6909730

[103] Maddalena A , Tornabene P , Tiberi P , et al . Triple vectors expand AAV transfer capacity in the retina. Mol Ther 2018;26:524–41. 10.1016/j.ymthe.2017.11.019 29292161 PMC5835116

[104] Dyka FM , Molday LL , Chiodo VA , et al . Dual Abca4-AAV vector treatment reduces pathogenic retinal A2E accumulation in a mouse model of Autosomal recessive stargardt disease. Hum Gene Ther 2019;30:1361–70. 10.1089/hum.2019.132 31418294 PMC6854433

[105] Piotter E , McClements ME , MacLaren RE . Therapy approaches for stargardt disease. Biomolecules 2021;11:1179. 10.3390/biom11081179 34439845 PMC8393614

[106] Siles L , Ruiz-Nogales S , Navinés-Ferrer A , et al . Efficient correction of Abca4 variants by CRISPR-Cas9 in hiPSCs derived from stargardt disease patients. Molecular Therapy - Nucleic Acids 2023;32:64–79. 10.1016/j.omtn.2023.02.032 36969552 PMC10034418

[107] Leibowitz ML , Papathanasiou S , Doerfler PA , et al . Chromothripsis as an on-target consequence of CRISPR-Cas9 genome editing. Nat Genet 2021;53:895–905. 10.1038/s41588-021-00838-7 33846636 PMC8192433

[108] Papathanasiou S , Markoulaki S , Blaine LJ , et al . Whole chromosome loss and genomic instability in mouse embryos after CRISPR-Cas9 genome editing. Nat Commun 2021;12:5855. 10.1038/s41467-021-26097-y 34615869 PMC8494802

[109] Ochmann MT , Ivics Z . Jumping ahead with sleeping beauty: mechanistic insights into cut-and-paste transposition. Viruses 2021;13:76. 10.3390/v13010076 33429848 PMC7827188

[110] Hernandez M , Recalde S , Garcia-Garcia L , et al . Preclinical evaluation of a cell-based gene therapy using the sleeping beauty transposon system in choroidal neovascularization. Mol Ther Methods Clin Dev 2019;15:403–17. 10.1016/j.omtm.2019.10.013 31890733 PMC6909167

[111] Li R , Zhuang Y , Han M , et al . PIggyBac as a high-capacity transgenesis and gene-therapy vector in human cells and mice. Dis Model Mech 2013;6:828–33. 10.1242/dmm.010827 23519027 PMC3634665

[112] Bitoque DB , Fernandes CF , Oliveira AML , et al . Strategies to improve the targeting of retinal cells by non-viral gene therapy vectors. Front Drug Deliv 2022;2. 10.3389/fddev.2022.899260

[113] Saha S , Woodard LE , Charron EM , et al . Evaluating the potential for undesired genomic effects of the piggybac transposon system in human cells. Nucleic Acids Res 2015;43:1770–82. 10.1093/nar/gkv017 25605795 PMC4330379

